# Intraguild Interactions Among Natural Enemies in the Trophic Web of *Bemisia tabaci* (Hemiptera: Aleyrodidae) on Melons

**DOI:** 10.3390/insects16080838

**Published:** 2025-08-14

**Authors:** Elena López-Gallego, Luis Gabriel Perera-Fernández, María José Ramírez-Soria, Juan Antonio Sanchez

**Affiliations:** Biological Control and Ecosystem Services Laboratory, Instituto Murciano de Investigación y Desarrollo Agrario y Medioambiental, C/Mayor s/n, La Alberca, 30150 Murcia, Spain; luisg.perera@carm.es (L.G.P.-F.); mjramirezsoria@gmail.com (M.J.R.-S.); juana.sanchez23@carm.es (J.A.S.)

**Keywords:** biological control, *Deraeocoris serenus*, *Eretmocerus eremicus*, intraguild interactions, *Orius laevigatus*, parasitoids, predators

## Abstract

*Bemisia tabaci* is a major pest of open-field melons in Mediterranean regions. Combining several species of natural enemies can improve pest control but it can give undesirable results when the species engage in antagonistic interactions. This study evaluated the effectiveness of *Deraeocoris serenus, Orius laevigatus,* and *Eretmocerus eremicus* as biological control agents and the interactions among the species. This was assessed with the presence–absence of all three species combined in a full-factorial assay. Each microcosm consisted of a melon plant grown in a 5 L pot enclosed in fine mesh, with each of the eight treatments replicated four times. Whiteflies were counted on three leaves per plant, and natural enemies were counted on the whole plants over six weeks. Basal leaves were taken to the laboratory and observed for the emergence of parasitoids. All three natural enemies independently significantly reduced whitefly numbers. *Deraeocoris serenus* was the most effective biocontrol agent, achieving whitefly reductions of more than 100-fold compared to the control. In contrast, *O. laevigatus* and *E. eremicus* achieved reductions of approximately 2.4-fold and 3.6-fold, respectively. No additive or synergistic effects in whitefly control were observed when two or more natural enemies were combined. *Deraeocoris serenus* and *O. laevigatus* were found to engage in mutual antagonistic interactions, and these two predators also interfered with the parasitoid. In addition, *E. eremicus* was also found to interfere with *D. serenus*. Understanding such interactions is essential for optimizing the management of natural enemies and improving biological pest control.

## Simple Summary

The whitefly *Bemisia tabaci* is a major pest of melon crops in Mediterranean regions. Natural enemies may control this pest, but antagonistic interactions may reduce their effectiveness. This research evaluated the combination of three biocontrol agents to determine the effect on whitefly control and their interactions. No additive effect on whitefly control was observed. The effectiveness of *Deraeocoris serenus* was much higher than that of *Orius laevigatus* and *Eretmocerus eremicus*. All three species engaged in antagonistic interactions. These findings highlight the importance of understanding interactions among natural enemies to improve biocontrol.

## 1. Introduction

Agroecosystems are characterized by a diversity of natural enemy species, which exhibit a wide range of sizes, mobility, and feeding habits, forming complex food webs that lead to variable results in pest control [1]. Although the presence of a diverse array of natural enemies with different hunting mechanisms could have an additive effect on pest control within an agroecosystem [2], this does not necessarily lead to optimal pest control outcomes, as their interactions may disrupt each other’s specific activities [3,4]. Natural enemies can participate in intraspecific predation (cannibalism) or interspecific predation, whether intra- or extra-guild (classical predation) [1]. Consequently, the impact on pest control is determined not only by the presence of natural enemies but also by their interactions [5]. These interactions are influenced by several factors, including feeding strategies, size, and mobility [6,7,8], attack rate [9,10], behavior [11,12], population structure, environmental complexity or habitat structure [1,13,14,15,16,17,18,19], and even the initial pest density [20].

One of the most relevant negative interactions is intraguild predation (IGP), which occurs when two or more species that share the same target engage in a trophic interaction with each other (parasitism or predation) [21]. The IGP model involves three or more species: the intraguild (IG) predator, the IG prey, and the shared prey. The IG prey feeds exclusively on the shared prey, whereas the IG predator feeds on both the IG prey and the shared prey [22]. IGP can be bidirectional, whereby both natural enemies attack each other, or unidirectional, whereby only one species attacks the other [5,21,23,24]. Accordingly, the collective impact of coexisting natural enemies on biological control has been observed to manifest in a range of outcomes, including synergistic [25], neutral, and antagonistic effects [26].

The sweet potato whitefly, *Bemisia tabaci* Gennadius (Hemiptera: Aleyrodidae), poses a significant threat to outdoor melons (*Cucumis melo* L.- Cucurbitaceae) in the southeastern region of the Iberian Peninsula [27]. *Bemisia tabaci* is a polyphagous pest that causes direct damage to plants and serves as a vector for viruses that severely impact plant health [28,29]. *Bemisia tabaci* exhibits a broad host range and considerable tolerance to elevated temperatures, which facilitate its dispersal to novel territories, rendering it challenging to control [30]. Furthermore, it has been demonstrated that this pest is prone to developing resistance to a diverse range of insecticides [30]. Consequently, there is growing interest in the development of alternative control management strategies and integrated pest management in the context of biological control.

Diverse natural enemies, including predatory Hemiptera (Anthocoridae, Miridae), and parasitoids (Aphelinidae, Encyrtidae), have been observed to attack *B. tabaci* in a wide range of crops [30,31]. Nevertheless, the potential of these natural enemies in biological control has only been examined for a few species, and mostly in laboratory conditions [31,32,33]. In Mediterranean melons, native natural enemies have been proved to play an important role in regulating pest populations [34,35]. Parasitoid species of the genus *Eretmocerus* (Hymenoptera: Aphelinidae) are considered the most efficient natural enemies of *B. tabaci* under variable climatic and agronomic conditions [24,33,36]. Additionally, *Orius* spp. (Hemiptera: Anthocoridae) and *Deraeocoris serenus* Douglas & Scott (Hemiptera: Miridae) are polyphagous species that have the potential to effectively reduce whitefly populations [31,37,38,39]. These generalist predators have been observed to coexist naturally in open-field melon crops when the pressure exerted by chemical treatments is reduced [34,35]. Nevertheless, there have been no studies seeking to elucidate the outcome of whitefly biological control when different species of these natural enemies are present simultaneously in the crop.

Predators and parasitoids that share a common prey or host are frequently found co-occurring in agroecosystems, where they can significantly influence the effectiveness of biological control programs through antagonistic interactions [5,40]. Understanding these interactions is essential for optimizing the combined use of natural enemies. In this research, we evaluated the efficacy of two predatory hemipterans, *Orius laevigatus* (Fieber) and *D. serenus*, along with the parasitoid *Eretmocerus eremicus* (Rose and Zolnerowich), in controlling *Bemisia tabaci*. Specifically, we aimed to examine the interactions among these three species and their collective impact on whitefly suppression. We hypothesized that (1) each natural enemy would significantly reduce *B. tabaci* populations when acting alone; (2) the combination of two or more natural enemies would lead to enhanced suppression; (3) antagonistic interactions would occur among these species; and (4) such interactions would influence the overall efficacy of whitefly control.

## 2. Materials and Methods

### 2.1. Plant and Insect Cultures

The specimens of *D. serenus* used in the experiment were originally collected in 2015, from a population in Torre Pacheco, southeastern Murcia, Spain (37°46′02″ N, 0°53′54″ W), on open field melons. The initial population size was 45 individuals. Taxonomic identification was conducted using the Goula key [41]. This predator was reared in plastic containers (26.5 × 17.5 × 12 cm) with two upper ventilation holes of 7.5 cm in diameter covered with a muslin mesh and fed on the eggs of *Ephestia kuehniella* Zeller (Lepidoptera: Pyralidae) ad libitum. Several small containers with cotton soaked with water were provided for hydration. The population of *D. serenus* in the laboratory ranged from 250 to 400 individuals. The colony was renewed annually by introducing twenty new individuals collected from the same area. *Orius laevigatus* and *E. eremicus* pupae were provided by Biobest N.V. (Westerlo, Belgium) a few days before their incorporation into the trial. *Orius laevigatus* were raised similarly to *D. serenus* until they reached adulthood. At the same time, *B. tabaci* adults were obtained from a colony reared on aubergine at IMIDA, Murcia. The founder adults used to establish this colony were supplied by Koppert España (La Mojonera, Almería, Spain) and were previously identified as the Q biotype (*B. tabaci* Mediterranean species) [42]. All insect colonies were reared and maintained in independent climatic chambers at 25 ± 1 °C and 60 ± 10% RH, with a 16L:8D photoperiod until inclusion in the assay. Melon plants of the Mural variety (toadskin type) provided by Syngenta España (Madrid, Spain), were cultivated in a climatic chamber in the same way mentioned above for insect breeding until they reached approximately 60 cm in height, with no pesticide application. After each sampling, the lateral shoots of the melon plants were removed to maintain the linear shape of the plant during the test.

### 2.2. Experimental Design

The trial was conducted following a complete factorial design, whereby the combination of three natural enemy species was tested at two levels (presence/absence), with whitefly always present. The treatments were as follows: (1) *D. serenus*; (2) *O. laevigatus*; (3) *E. eremicus*; (4) *D. serenus* and *O. laevigatus*; (5) *D. serenus* and *E. eremicus*; (6) *O. laevigatus* and *E. eremicus*; (7) *D. serenus*, *O. laevigatus* and *E. eremicus*; and (8) without natural enemies (control). The experiment was conducted within a compartment, thereby creating a microcosm. Each microcosm consisted of a 5 L plastic pot containing a melon seedling held vertically with raffia thread and enclosed in a 1.5 m high muslin bag. The muslin bag was equipped with a vertical zip to facilitate sampling and with an elastic band at the top and bottom to reduce the entry and exit of arthropods. The microcosms were placed within a greenhouse to affix the upper raffia thread to their structure and ensure that the melon plants remained upright. The greenhouse was equipped with a transparent plastic roof and perimeter mesh but lacked integrated systems for temperature and humidity control. After four days of plant acclimatization, 10 adult whiteflies per plant, aged between three and four days, were first released into each microcosm. A second identical release of whiteflies was carried out four days later. The natural enemies were released twenty-three days after the first introduction of whiteflies. Ten adult specimens of *O. laevigatus* and *D. serenus*, 2–3 days old (5 males and 5 females), and a commercial cardboard unit containing 100 pupae of the parasitoid *E. eremicus* were introduced. Prior to the trial, the emergence rate of *E. eremicus* was evaluated under the same environmental conditions using 16 cardboards, each containing 100 pupae, resulting in a 50% emergence rate and a sex ratio of 1.5 males per female. The cardboard was attached to the end of a rod with a silicone dot on the holder. The rod was impregnated with petroleum jelly to prevent direct contact with the plant and other system elements and inserted into the potting soil. Each microcosm contained a distinct combination of natural enemies, with *B. tabaci* as prey/host. Four replicates were conducted per treatment. The arrangement of the microcosms within the greenhouse was random. A data logger (HOBO^®^ Pro v2 Ext Temp/RH, Onset Computer, Bourne, MA, USA) was installed in the middle of the greenhouse to monitor the temperature and relative humidity. The average daily temperature over the period of the experiment was 26.7 °C, and the average minimum and maximum temperatures were 16.6 and 37.6 °C, respectively.

### 2.3. Sampling

Two samplings were carried out before the introduction of natural enemies on 9 and 16 May 2017 to verify the homogeneity of the whitefly establishment. In this preliminary study, the total number of nymphs and adults observed on the first 10 leaves of the melon plant was recorded. Following the second sampling, natural enemies were introduced (16 May). Five rounds of insect sampling were conducted weekly from 25 May to 22 June 2017. All the arthropods were counted using hands-free magnifying glasses (1.8–4.8×) on the second fully developed leaf from the apex, an intermediate leaf, and a basal leaf. Whitefly nymphs were considered predated when they appeared empty, without having completed their development. The parasitized nymphs could not be counted in the field due to the variety of developmental stages and because their identification required observing a visible spot through the cuticle under a stereomicroscope. To enable this, a basal leaf was removed, placed in a sealed plastic bag, and transported to the laboratory. The area of the leaf that was removed constituted an average sampled area of approximately 5% of the total. The petioles of the sectioned leaves were placed in a plastic container with water and then placed inside a 2 L plastic transparent cylinder measuring 24.5 × 11.5 cm with a 7.5 cm diameter hole at the top that was covered with a mesh for ventilation. These containers were kept in a climate chamber at 25 ± 1 °C and 60 ± 10% RH, with a 16L:8D photoperiod, and were checked weekly for the emergence of parasitoids over 30 days.

### 2.4. Data Analysis

Generalized linear mixed models (GLMMs) were used to test for the effect of natural enemies (*D. serenus*, *O. laevigatus*, and *E. eremicus*) and their interactions on the number of whiteflies alive (nymphs and adults) and predated (nymphs), expressed as the sum of the number of individuals on the three sampled leaves (an apex, an intermediate, and a basal leaf). The distribution of the experimental data was tested using “fitdist” (“fitdistrplus” package), and the choice of distribution function was based on the score of Akaike’s Information Criterion (AIC). The GLMMs were run with the “glmmPQL” function (“MASS” package ver. 7.3-61) using the negative binomial to explain the distribution of errors. The sampling date was introduced in the models as a random factor, and the models were diagnosed by assessing the distribution of the standardized residuals versus the fitted values. The χ^2^ and *p*-values for the fixed factors were obtained with the Wald test using the “Anova” function in the “car” package ver. 3.1-3. Pairwise comparisons between treatments were carried out using Tukey’s test with the function “glht” (“multcomp” package ver. 1.4-26). All the statistical analyses were carried out using R ver. 4.4.2 [43]. The same approach was followed to test for the interactions among the different species of natural enemies: (1) the effect of *O. laevigatus* and *E. eremicus* on the number of *D. serenus* per plant was tested; (2) the effect of *D. serenus* and *E. eremicus* on the number of *O. laevigatus* per plant was tested; and (3) the effect of *D. serenus* and *O. laevigatus* on the number of *E. eremicus* that emerged on the basal leaf in the laboratory was tested.

## 3. Results

### 3.1. Effect of D. serenus, O. laevigatus, and E. eremicus on the Abundance of B. tabaci

**Figure 1:**
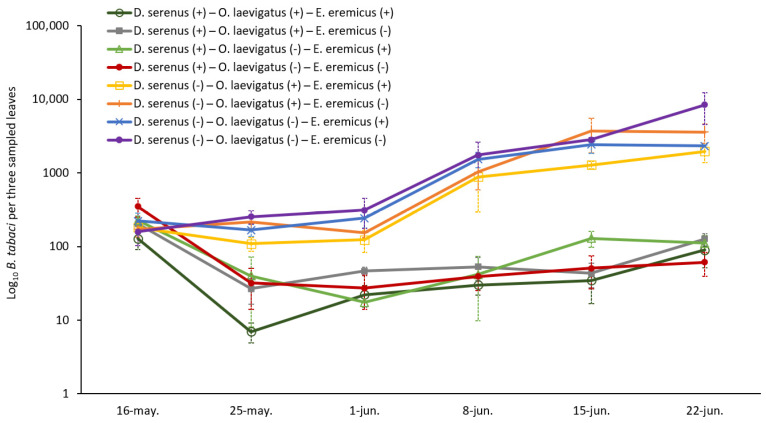
Average number of whiteflies ± SE (adults and nymphs) on the three sampled leaves (apex, middle, and basal) of melon plants in the different treatments throughout the sampling period. *Y*-axis values are log_10_-transformed. Note: (+) presence; (−) absence of the natural enemy.

The abundance of *B. tabaci* was significantly reduced in the presence of *D. serenus* (χ^2^(1) = 332.1, *p* < 0.001). In all treatments involving *D. serenus*, the whitefly population followed a similar trend (Figure 1). After the introduction of natural enemies, the whitefly numbers declined after the first sampling. Subsequently, the population progressively increased, reaching similar levels by the end of the experiment. Specifically, the average ± SE number of individuals summed across the three sampled leaves ranged from 7.0 ± 2.1 to 126.8 ± 21.8 (Figure 1). No significant differences were found among the treatments with *D. serenus* (*p* > 0.05), and all of them differed significantly from the control (*p* < 0.001).

The abundance of *B. tabaci* was also significantly reduced in the presence of *O. laevigatus* (χ^2^(1) = 7.6, *p* = 0.006). Treatments involving *O. laevigatus* showed similar population dynamics (Figure 1). At the end of the sampling period, the average (±SE) number of individuals summed across the three sampled leaves reached 3589.8 ± 1048.1 in the *O. laevigatus*-alone treatment and 1962.0 ± 583.7 in the *O. laevigatus*–*E. eremicus* treatment. No significant differences were detected between these two treatments (*p* > 0.05), but both differed significantly from the control (*p* < 0.001) and from all treatments where *D. serenus* was present (*p* < 0.001).

Finally, the abundance of *B. tabaci* was significantly reduced in the presence of *E. eremicus* (χ^2^(1) = 9.4, *p* = 0.002), with the abundance being significantly lower than the control (*p* < 0.001; Figure 1). At the end of the experiment, the whitefly reached 2321.5 ± 80.2 individuals (average ± SE number of individuals summed across the three sampled leaves) in the treatment where *E. eremicus* was alone and 8436.5 ± 3854.4 individuals in the control (Figure 1). No significant differences in the number of whiteflies were observed when *E. eremicus* was present alone or in combination with *O. laevigatus* (*p* > 0.05), but it differed significantly when combined with *D. serenus* (*p* < 0.001).

### 3.2. Interaction Between Natural Enemies

**Figure 2:**
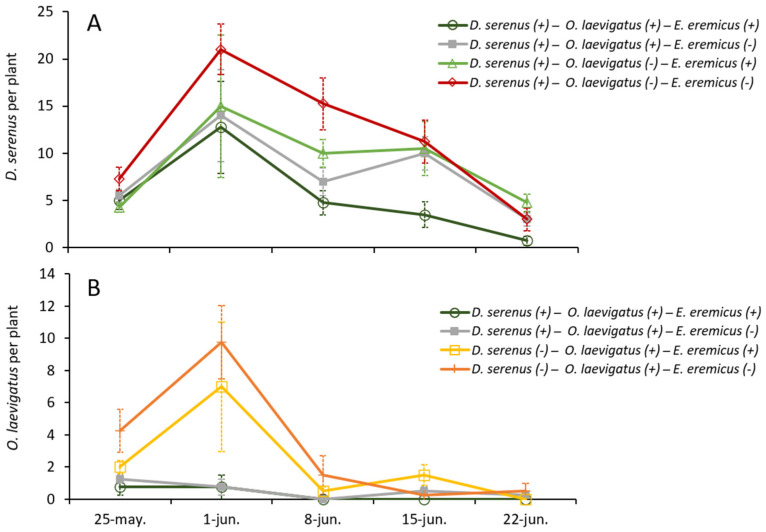
Average number per plant ± SE of (**A**) *D. serenus* in presence (+)/absence (−) of *O. laevigatus* and *E. eremicus*, and (**B**) *O. laevigatus* in the presence (+)/absence (−) of *D. serenus* and *E. eremicus*.

The abundance of *D. serenus* was significantly reduced in the presence of *O. laevigatus* (χ^2^(1) = 15.1, *p* < 0.001) as well as in the presence of *E. eremicus* (χ^2^(1) = 7.2, *p* = 0.007). Across all treatments involving *D. serenus*, peak abundances were recorded on the second week of the experiment and declined progressively thereafter until the end of the trial (Figure 2A). The highest peak of abundance was observed when *D. serenus* was assayed alone (21.0 ± 2.7 individuals per plant) and the lowest when the three species were assayed together (12.8 ± 4.9 individuals per plant) (Figure 2A).

The abundance of the anthocorid *O. laevigatus* was significantly reduced by *D. serenus* (χ^2^(1) = 27.3, *p* < 0.001; Figure 2B). In contrast, the abundance of *O. laevigatus* was not significantly reduced by *E. eremicus* (χ^2^(1) = 3.0, *p* = 0.082). In the treatment where *O. laevigatus* was assayed in the absence of *D. serenus*, both with and without *E. eremicus*, the abundance of *O. laevigatus* peaked in the second week of the assay and declined progressively until the end of the trial (Figure 2B).

**Figure 3:**
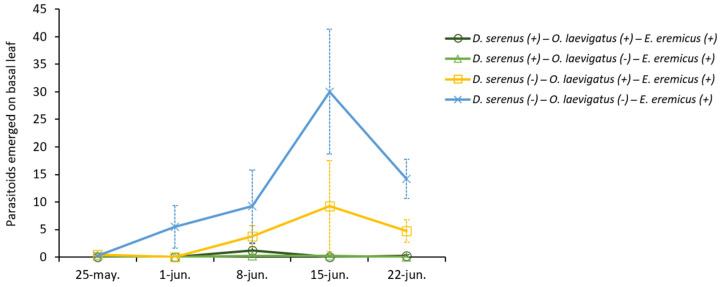
Average number ± SE of *E. eremicus* hatched from whitefly nymphs on basal melon leaves in the treatments where *E. eremicus* was released. (+) presence; (−) absence of the natural enemy.

The emergence of parasitoids in the leaves taken to the laboratory was significantly reduced by *D. serenus* (χ^2^(1) = 37.8, *p* < 0.001), whereas the effect of *O. laevigatus* was not found to be significant (χ^2^(1) = 2.6, *p* = 0.102). In the treatments without predators, the parasitoid emergence peaked at 30.0 ± 11.3 individuals (average ± SE) four weeks after release (Figure 3). In the treatment where *D. serenus* was present—either alone or in combination with *O. laevigatus*—the parasitoid emergence remained low throughout the trial, and no significant differences were observed between these two treatments (*p* > 0.05; Figure 3).

### 3.3. Impact of Natural Enemies on Whitefly Predation

**Figure 4:**
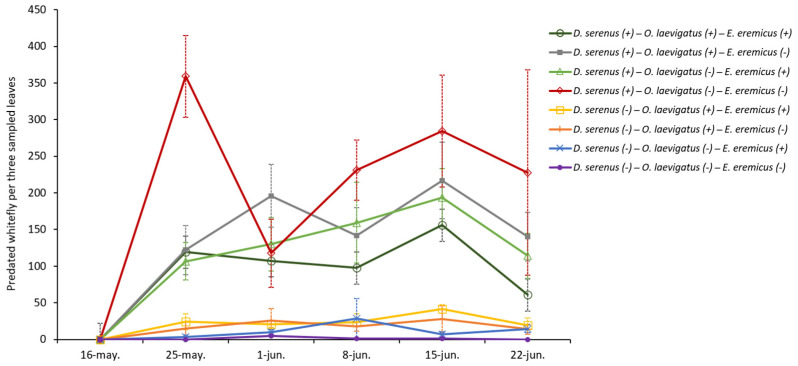
Average number ± SE of predated *Bemisia tabaci* nymphs on the three sampled leaves (apex, middle, and basal) of melon plants in each treatment throughout the sampling period. (+) presence; (−) absence of the natural enemy.

Predation on whitefly nymphs increased significantly in the presence of *D. serenus* (χ^2^(1) = 218.1, *p* < 0.001). Some of the interactions between predators and parasitoids also had a significant negative effect on *B. tabaci* predation: *D. serenus*–*O. laevigatus* (χ^2^(1) = 31.8, *p* < 0.001) and *D. serenus*–*E. eremicus* (χ^2^(1) = 23.0, *p* < 0.001). In addition, the three-way interaction among *D. serenus*, *O. laevigatus*, and *E. eremicus* was also found to have a significant effect on the predation of the whitefly (χ^2^(1) = 9.9, *p* = 0.002). Treatments involving *D. serenus* exhibited similar trends in whitefly predation, with no significant differences detected among them (*p* > 0.05; Figure 4).

In the presence of the predator *O. laevigatus*, predation on whitefly nymphs also increased significantly, albeit to a lesser extent than with *D. serenus* (χ^2^(1) = 9.2, *p* = 0.002). Treatments with *O. laevigatus* alone or in combination with *E. eremicus* exhibited similar predation dynamics, with no significant differences between them (*p* > 0.05; Figure 4). Finally, the parasitoid *E. eremicus* had no significant effect on whitefly predation (χ^2^(1) = 2.0, *p* = 0.156; Figure 4).

## 4. Discussion

This study assessed the interactions among several natural enemies of the *B. tabaci* guild (two predatory hemipterans–*D. serenus* and *O. laevigatus*–and one parasitoid, *E. eremicus*) and their impact on whitefly control on melon plants, both individually and in combination. In agreement with our first hypothesis, each of the natural enemies significantly reduced the whitefly populations when acting alone. Similar findings have been reported by other authors, who observed effective suppression of whitefly populations by *Deraeocoris* spp. [37,44], *Orius* spp. [39] and *Eretmocerus* spp. [45,46] in cotton, melons, tomatoes, and peppers. However, the impact of *D. serenus* on whitefly abundance was significantly greater than that of *O. laevigatus* or *E. eremicus*, aligning with its higher observed predation and possibly reflecting a greater capacity of the mirid to exploit this resource [42]. *Deraeocoris serenus* alone reduced the whitefly population by more than 138-fold compared to the control. In contrast, *O. laevigatus* and *E. eremicus* achieved reductions of approximately 2.4-fold and 3.6-fold, respectively. These results clearly highlight the superior efficacy of *D. serenus* for whitefly control. This could be explained by the greater size and more aggressive predatory behavior of this mirid in comparison to smaller natural enemies [6,9,47].

Experimental evidence indicates that increased diversity among natural enemies employing different hunting behaviors enhances prey suppression more effectively than groups of predators using similar attack strategies [2,48,49]. Consequently, combining predators and parasitoids would be expected to enhance the suppression of *B. tabaci* populations. For example, releases of the predator *Delphastus pusillus* (LeConte) (Coleoptera: Coccinellidae) in combination with the parasitoids *Encarsia* spp. (Hymenoptera: Aphelinidae) have resulted in greater reductions in whitefly populations compared to treatments involving parasitoid-only combinations [50]. Similarly, the most effective suppression of *B. tabaci* has been reported in greenhouse tomato systems using the combined action of *Eretmocerus mundus* Mercet (Hymenoptera: Aphelinidae) and *Macrolophus caliginosus* Wagner (Hemiptera: Miridae) [51]. Additionally, studies examining the coexistence of *Macrolophus pygmaeus* Rambur (Hemiptera: Miridae) and *Nesidiocoris tenuis* (Reuter) (Hemiptera: Miridae) alongside *E. mundus* have also demonstrated enhanced whitefly control [52]. In contrast, in the present study, no additive or synergistic effects were observed when *D. serenus*, *O. laevigatus*, and *E. eremicus* were combined, compared to treatments in which each species acted alone. These findings are consistent with those reported by Malo et al. [53], who observed no improvement in *B. tabaci* suppression when adults of *M. pygmaeus* and the parasitoid *E. mundus* were released simultaneously on tomato and cotton plants. In the present study, the lack of enhanced whitefly control when combining three natural enemies could be due to the dominant impact of the predator *D. serenus* on *B. tabaci*, which likely overshadowed the contribution of the other two natural enemies, and to the negative effect of the mirid on the anthocorid and the parasitoid and vice versa.

Intraguild predation (IGP), a widespread phenomenon in which predators and parasitoids sharing a common prey engage in interference, can reduce overall biological control efficiency through antagonistic interactions among natural enemies [5,54]. This may help explain the lack of synergistic effects observed in the present study. In this research, *D. serenus* caused a decline in the population of the anthocorid *O. laevigatus* when both predators coexisted. This observation aligns with the findings of Woodward and Hildrew [8], who noted that the coexistence of two generalist predators can lead to unidirectional IGP, whereby the larger predator exploits the smaller one. Interestingly, we also observed a reduction in *D. serenus* populations in the presence of *O. laevigatus*, suggesting a mutual negative interaction between these two IG predators. This reciprocal suppression points to the potential occurrence of bidirectional or symmetrical IGP, possibly involving the late-instar or adult stages of both species preying on the early developmental stages of one another. Thus, despite the larger size and voracity of *D. serenus*, its early instars remain vulnerable, resulting in interference when coexisting with the smaller predator *O. laevigatus* [17,21].

Furthermore, the reduced emergence of the parasitoid *E. eremicus* in the presence of *D. serenus* suggests that this predator interferes with the parasitism process—either by preying on parasitized whitefly nymphs or by reducing the availability of suitable hosts. On one hand, previous studies have commonly reported asymmetric interactions between *Eretmocerus* spp. and co-occurring generalist predators [24,55]. Such interference is typically characterized by unidirectional predation, in which predators attack parasitoids or parasitized hosts, while parasitoids lack the capacity to harm predators directly [56,57]. On the other hand, parasitism rates in *E. eremicus* have been found to decline as the number of *B. tabaci* nymphs decreased [58]. Additionally, predators have been shown to disrupt parasitoid foraging behavior, thereby reducing their biological control efficacy [54,59,60].

Surprisingly, in this research *E. eremicus* was found to have a negative effect on *D. serenus*. This effect is unlikely to have been driven by exploitative competition, since a similar whitefly decline was observed in all the treatments where *D. serenus* was involved and prey was always relatively abundant. Increased habitat-leaving rates due to the reduction in patch quality could explain the low number of mirids on plants shared with the parasitoids [61]. *Deraeocoris serenus* could reject whitefly nymphs parasitized or fed by the parasitoid due to changes in host quality or chemical cues [62]. Alternatively, interference through behavioral or chemical means—such as the disruption of foraging or spatial displacement—could underlie the observed negative effect [53,63,64]. Similar interactions between parasitoids and predators have been reported in systems where host quality or predator behavior is influenced by parasitism rather than host availability alone [5,65]. In contrast, *E. eremicus* was not found to interfere with *O. laevigatus*. This could be explained by the vertical stratification of whitefly pre-imaginal stages within the plant canopy: eggs and early instars typically occur on apical leaves, while later stages are found on older, basal leaves [66]. As a result, *O. laevigatus* forages predominantly in the upper canopy, where immature whiteflies are concentrated, whereas *E. eremicus* targets later-stage nymphs in lower canopy layers [36,67]. This spatial segregation may reduce direct encounters and facilitate coexistence.

Antagonistic interactions can significantly influence whitefly suppression by altering the dynamics among natural enemies [56,62,68,69]. In the present study, the combined presence of the IG predators *O. laevigatus* and *D. serenus* resulted in reduced predation on whitefly nymphs. This finding aligns with the observations of Moreno-Ripoll et al. [70], who reported that females of the mirids *M. pygmaeus* and *N. tenuis* engaged in IGP and cannibalism under laboratory conditions, ultimately diminishing their collective impact on *B. tabaci*. In contrast, Lucas and Alomar [71] found that the combination of *M. caliginosus* and *Dicyphus tamaninii* Wagner (Hemiptera: Miridae) did not disrupt predation; instead, their joint presence enhanced whitefly suppression relative to single-predator scenarios. Such discrepancies in interspecific outcomes likely reflect the identity of the natural enemies involved, the experimental scale and conditions, the availability of shared or alternative prey, and the duration of exposure [6,52,72,73]. Although theoretical frameworks suggest that combining natural enemies with differing hunting strategies should enhance prey suppression [48,49], our results indicate the opposite: predation on whitefly nymphs decreased when either predator (*D. serenus* or *O. laevigatus*) coexisted with the parasitoid *E. eremicus*. These findings contrast with those of Bao-Fundora et al. [74], who reported increased predation by *Geocoris punctipes* (Say) (Hemiptera: Geocoridae) when combined with *E. eremicus*, despite the occurrence of IGP. In the present study, the simultaneous presence of both IG predators and the IG prey (*E. eremicus*) resulted in reduced predation, yet the overall whitefly suppression remained comparable to that observed in individual enemy treatments. This outcome is partially consistent with the work of Moreno-Ripoll et al. [52], who found that the coexistence of mirid predators (*M. pygmaeus* and *N. tenuis*) with the parasitoid *E. mundus* reduced the predation pressure on *B. tabaci*; however, unlike our findings, their combined presence ultimately enhanced whitefly control. In contrast, the lack of an additive effect in our study may be attributed to the strong individual impact of *D. serenus*, whose dominant predatory role likely overshadowed or disrupted the contribution of additional natural enemies through IG interactions. These findings are consistent with the theoretical framework proposed by Schmitz [75], who noted that the combined impact of multiple predators on a shared prey species is often equivalent to, or even less than, that of a single predator due to interference and emergent interspecific interactions.

In conclusion, this study shows that combining two predators (*D. serenus* and *O. laevigatus*) and one parasitoid (*E. eremicus*) gave similar whitefly control in melon plants compared to using just one highly effective predator, such as *D. serenus*. *Orius laevigatus* was observed to be a much less efficient whitefly predator than *D. serenus*, and combining it with the parasitoid did not improve whitefly control either. *Deraeocoris serenus* and *O. laevigatus* were found to engage in mutual antagonistic interactions. Curiously, *D. serenus* and *E. eremicus* were also found to mutually interfere. Some of the interactions among natural enemies were observed to have a significant effect on whitefly predation. Given the importance of the findings of this study from an economic and effectiveness point of view, the interactions among these natural enemies should be taken into account in biological pest control programs. Future research should determine whether these IG interactions persist under open-field conditions or are mitigated by factors such as food diversity, increased habitat complexity, etc. Ultimately, whitefly control in unconfined environments is likely to depend on the composition of natural enemy communities and multiple ecological variables. A comprehensive understanding of these interactions is required for optimizing the success of biological control strategies.

## Data Availability

The analyzed and generated datasets will be maintained at the IMIDA repository and will be available upon request. Voucher specimens have been preserved in the insectary of IMIDA to confirm their identity in case future verification is required.
